# Low cost additive manufacturing of microneedle masters

**DOI:** 10.1186/s41205-019-0039-x

**Published:** 2019-02-04

**Authors:** Ashley R. Johnson, Adam T. Procopio

**Affiliations:** Merck & Co., Inc, Kenilworth, NJ USA

**Keywords:** Drug delivery, Microneedles, Sterolithography, 3D printing, Aliasing

## Abstract

**Purpose:**

Microneedle patches are arrays of tiny needles that painlessly pierce the skin to deliver medication into the body. Biocompatible microneedles are usually fabricated via molding of a master structure. Microfabrication techniques used for fabricating these master structures are costly, time intensive, and require extensive expertise to control the structure’s geometry of the structure, despite evidence that microneedle geometry is a key design parameter. Here, a commercially available 3D printer is utilized, for the first time, to quickly and easily manufacture microneedle masters.

**Design/methodology/approach:**

Because commercially available 3D printers are not typically used for micron-scale fabrication, the influence of three different sources of error- stair-stepping, aliasing, and light abberations- on the resulting structure is investigated. A custom Matlab code is written to control the light intensity projected off of each individual micromirror (through grayscale) at a given time. The effect of the layer height, the number of layers, and grayscale on the sharpness, surface texture, and dimensional fidelity of the final structure is described.

**Findings:**

The Autodesk Ember is successfully utilized to fabricate sharp microneedles with a tip radius of approximately 15 μm in less than 30 min per patch (as compared to weeks to months for existing approaches). Utilization of grayscale improves surface texture and sharpness, and dimensional fidelity within ±5% of desired dimensions is achieved.

**Originality/value:**

The described 3D printing technique enables investigators to accurately fabricate microneedles within minutes at low cost. Rapid, iterative optimization of microneedle geometry through 3D printing will accelerate microneedle research through improved understanding of the relationship between microneedle structure and function.

**Electronic supplementary material:**

The online version of this article (10.1186/s41205-019-0039-x) contains supplementary material, which is available to authorized users.

## Introduction

Microneedles are arrays of sub-millimeter sized needles that painlessly pierce the outer layer of the skin to deliver medicine into the body [1–3]. Microneedles are particularly useful for the delivery of molecules that cannot be delivered orally, such as proteins, peptides, and molecules with poor solubility or permeability [1–3]. Because drug delivery using pain free microneedles is preferred by patients as compared to hypodermic needles, microneedle based delivery may be an attractive commercial product exhibiting improved patient compliance, particularly for indications requiring frequent injections, such as insulin or hormone therapies [4, 5].

Fabrication of polymeric microneedles is usually accomplished through silicone molding of a master structure. Master structures are commonly fabricated using silicon based manufacturing techniques including wet etching, dry etching, or photolithography [6–8]. Microneedle size and shape dictate penetration ability, drug loading capacity and release rates,but extensive optimization is required to control geometry with existing approaches. Design constraints, such as limited available aspect ratios, are often introduced due to technical limitations of the fabrication technologies [9]. Further, the costly equipment and complex processes required [6, 10] result in long lead times (on the order of months) and present a high barrier to entry into the microneedle field.

More recently, additive manufacturing (“3D Printing”) of microneedle arrays has been investigated. Additive manufacturing enables nearly unlimited control over microneedle design. Stereolithography, a conventional 3D printing approach that fabricates parts by shining light onto a photoreactive resin, has been used to fabricate microneedles, but with suboptimal fidelity to the desired structure [11, 12]. For example, Lu et al. demonstrate stereolithography of microneedles, but the needles appear to be visually blunt [11]. Alternatively, Narayan and colleagues have utilized a high resolution 3D printing technique called two photon polymerization to fabricate microneedle arrays [13]. While this approach produces ultrasharp microneedle arrays, fabrication times are long (on the order of days, due to the time required to raster the laser over the entire array) and access to costly and highly specialized equipment is required. Johnson et al. demonstrated rapid fabrication of sharp microneedle arrays using a new continuous 3D printing technique called Continuous Liquid Interface Production (CLIP) in less than two minutes per array [14], but the high resolution equipment that was used is not currently commercially available (though lower resolution systems are on the market). Therefore, the ability to 3D print microneedle masters quickly using commercially available equipment would be an improvement in the field.

**Fig. 1:**
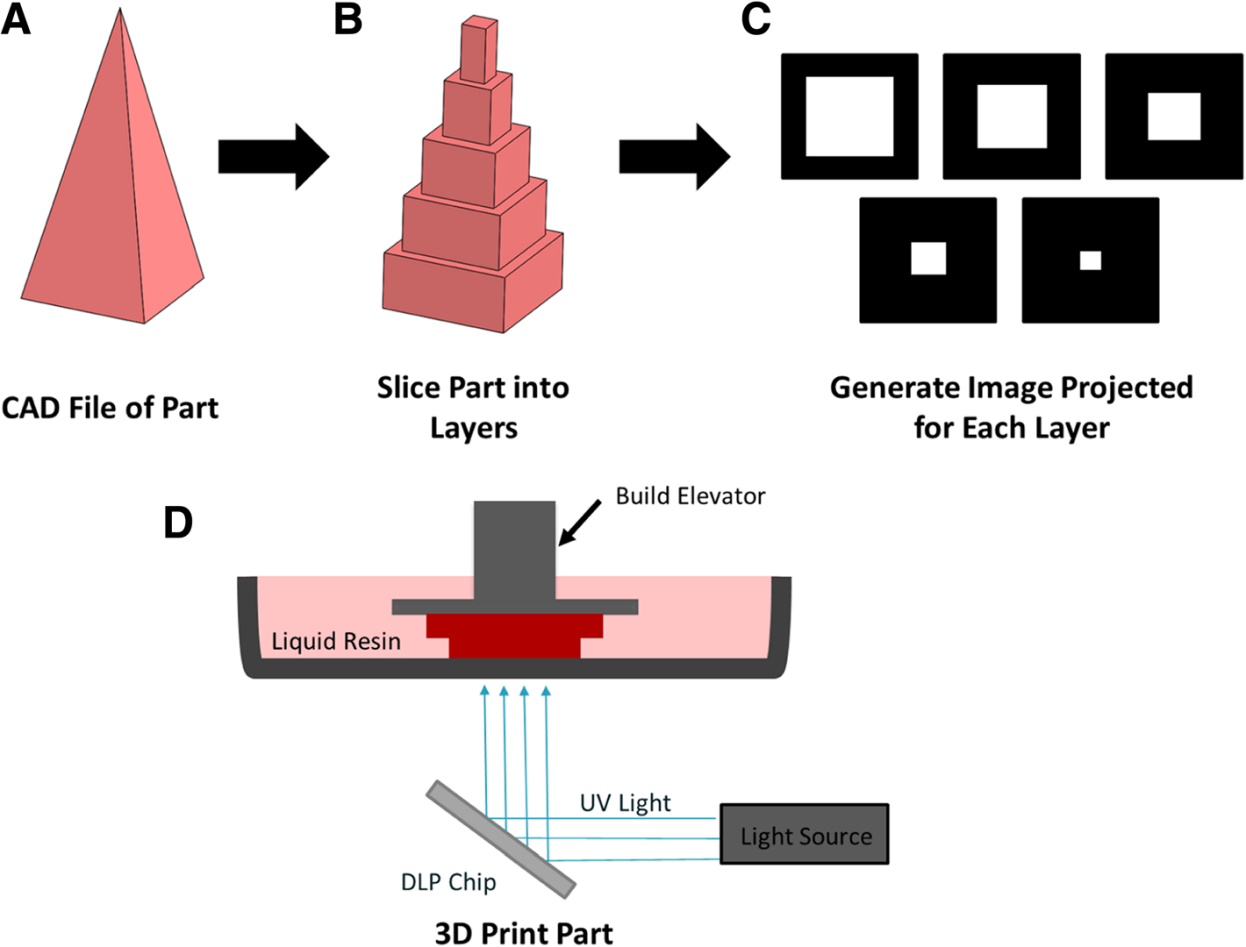
The stereolithography process. **a**) A CAD file of the desired part is created and **b**) computationally sliced into layers. **c**) Each layer is converted to an image that is **d**) projected into the resin bath as the layer is being fabricated. The image is generated by reflecting a light off of an array of micromirrors called a DLP chip and into the resin. Light induced polymerization produces the part

In this report, we demonstrate additive manufacturing of microneedle arrays using the Autodesk® Ember™, a commercial desktop 3D printer which retailed for approximately $7500 USD at the time of experimentation. This stereolithographic 3D printer produces parts one layer at a time by photopolymerization (light induced polymerization) (Fig. 1). First, a computer aided design (CAD) file of the desired part is created and computationally sliced into cross-sections (layers) (Fig. 1a-b). A digital light projection (DLP) chip then projects an image of each layer into the resin bath. When the light image reaches the resin bath, photopolymerization cures the part in a layer by layer fashion (Fig. 1c).

**Fig. 2:**
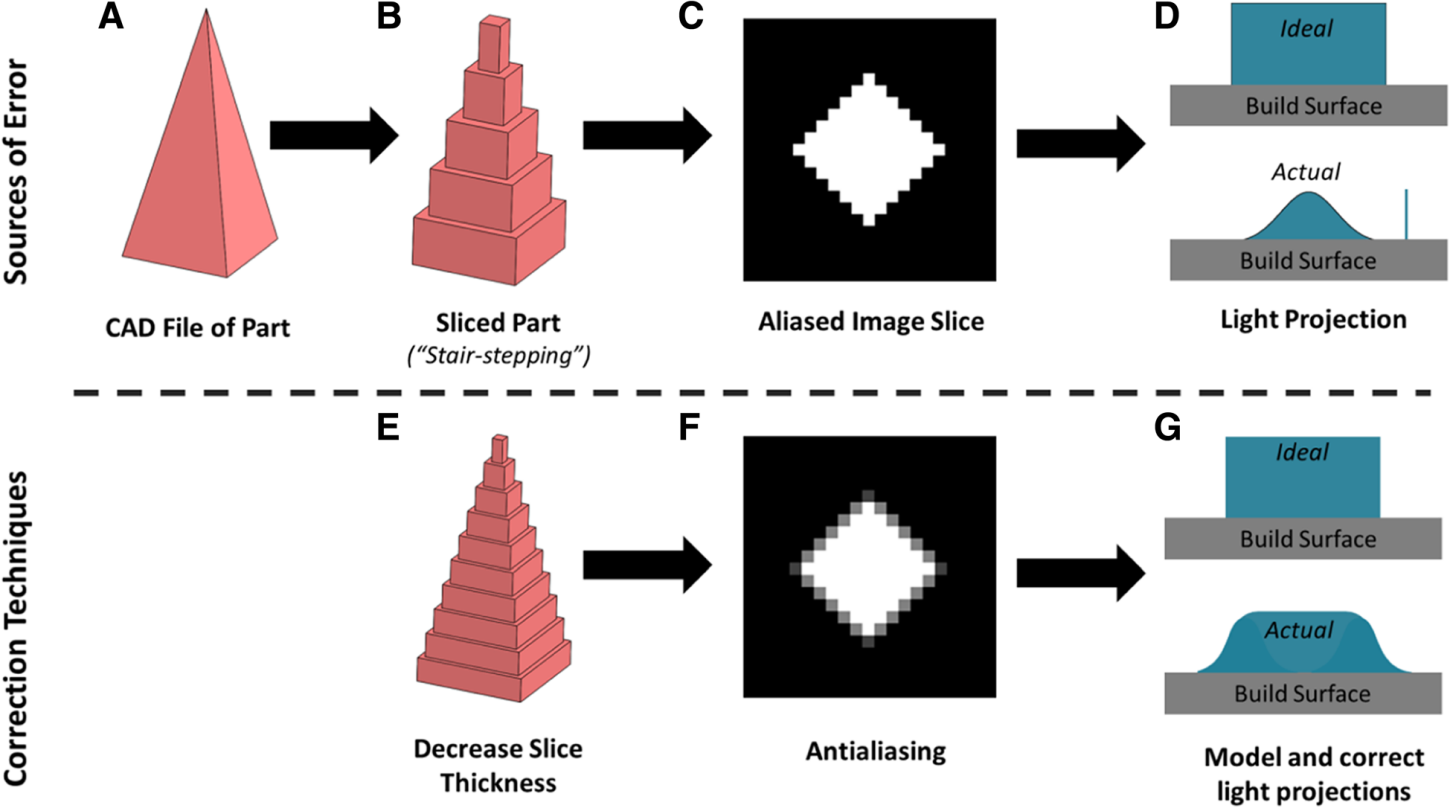
Sources of error and correction techniques in stereolithography based 3D printing Process. **a** The 3D printing process begins with a CAD file of the desired part. The part is then **b**) computationally sliced into layers which are an imperfect approximation of the original part. **c**) Each layer is then converted into an image that can be projected off of a DLP chip. Because the images are generated using a discrete array of pixels, the image is also an approximation (alias) of the desired object with jagged edges. **d** When the image reaches the build surface, diffraction and imperfect focus distort the image. These sources of error can be corrected by **e**) decreasing the thickness of each slice, **f**) improving image resolution by introducing grayscale (antialiasing) and **g**) modeling and correcting light distributions (often also employing grayscale). Note that the images in **c**) and **f**) show the microneedle being diagonally oriented relative to the DLP chip to exaggerate aliasing for visualization. In reality, images are oriented orthogonally to the DLP chip, as shown in Fig. 1, Additional file 5: Figure S5, Additional file 6: Figure S6 and Additional file 7: Figure S7

Stereolithography based 3D printers are known to introduce three types of defects into parts (Fig. 2). “Stair-stepping” is introduced when a continuous part is sliced into layers in the z direction. The resulting stack of layers is an imperfect approximation of the original part with jagged surfaces (Fig. 2b). A second type of defect, “aliasing”, is produced when each layer is projected as an individual image. Each image is projected off of a DLP chip- an array of micromirrors which produces the image by reflecting light off of individual micromirrors in either the “ON” or the “OFF” position. Each individual micromirror makes up a single 50 × 50 μm pixel within the light image, where black pixels are micromirrors in the “OFF” position and white pixels are micromirrors in the “ON” position. Because the DLP chip contains a finite number of pixels, the projected image is an approximation of each layer in the part, with the resolution defined by the number of micromirrors in the array (Fig. 2c). Lastly, additional errors are introduced due to imperfect focus of the image and diffraction of light within the 3D printer. These aberrations introduce differences between the image sent to the projector and the light distribution that reaches the resin (Fig. 2d) [15].

Several techniques have been investigated to mitigate these defects. For example, “stair-stepping” can be mitigated by decreasing layer height [16, 17]. This approach improves the fidelity of the part to the desired CAD file, but increases fabrication time due to realignment between steps (Fig. 2e). In order to reduce “aliasing”, a technique called “anti-aliasing” is used [18, 19]. “Anti-aliasing” improves the resolution of each projected image by adding grayscale into the black and white images (Fig. 2f). Physically, this grayscale is manifested by rapidly dithering the micromirror on the DLP chip such that the mirror alternates between the “ON” (white) and “OFF” (black) positions at a high frequency. The percentage of time spent in the “ON” position determines the amount of light that is projected, which controls the volume of resin that polymerizes to form the part. Therefore, anti-aliasing (introducing grayscale) provides more levels of control over which resin cures to produce a smoother part. Lastly, a number of (slightly more complicated) techniques have been used to correct for errors caused by aberration of light. For example, several authors have modeled light intensity distributions created using DLP chips in order to optimize the light intensity projected off of each pixel to most closely match the desired CAD file (Fig. 2g) [15, 20].

In this work, we investigate using the Autodesk® Ember™ to fabricate microneedle masters. We study the effect of each type of defect (“stair-stepping”, “aliasing” and light effects) on the resulting microneedle master structure. We find that with proper use of correction techniques the Autodesk® Ember™ is capable of producing sharp microneedle arrays in less than 30 min per patch. We anticipate that additive manufacturing of microneedle arrays will lower the barrier to entry into the microneedle field for researchers with no background in microfabrication. Further, the ability to easily adjust microneedle size and shape can reduce lead times for new microneedle designs, catalyzing the optimization of microneedle arrays in preclinical research. This ability to rapidly iterate microneedle geometry may enable improvements in maximum drug loading, reductions in the required force of application, or an increase in the strength of microneedles in an array [10].

## Materials and methods

### Microneedle fabrication using default settings

Square pyramidal microneedle arrays measuring 1000 μm tall and 450 μm in width, spaced at 450 μm between needles (edge to edge) were designed using Solidworks 2016. The array was 12 × 12 microneedles, for a total of 144 microneedles per patch. The margin from the last microneedle row to the edge of the array was 450 μm in all directions (Additional file 1: Figure S1). This computer aided design file was exported in a Standard Triangle Language (.STL) format, imported into Autodesk’s Print Studio software, centered on the build area with microneedles oriented along the z axis and exported as a .tar.gz file without introducing additional supports. Layer thicknesses of 10 μm, 25 μm, and 50 μm were investigated. The file was uploaded to the Autodesk Ember and fabricated using Autodesk’s Standard Clear PR48 Resin (formulation given in Additional file 2: Figure S2). All microneedle arrays were imaged without sputter coating using an FEI Quanta 200 electron microscope (Hillsborough, Oregon, United States) in low vacuum mode at 0.028 Torr, 20.0 kV, and 3.0 spot size. Microneedle physical dimensions (height, width, tip radius of curvature) were measured using Image J (National Institutes of Health, Bethesda, Maryland, USA). Measurements were taken from three needles at random locations on two separate arrays.

### Analysis and alteration of antialiasing and image slices

In order to analyze the individual images projected for each slice, .tar.gz files created by PrintStudio were unzipped with 7-zip file manager (Igor Pavlov, version 9.22 beta). Individual Portable Network Graphic (.PNG) image slices were analyzed to determine which micromirrors were assigned to be “ON” (white), “OFF” (black) or dither in each projection.

In order to investigate the influence of aliasing on microneedle structure, we wrote a proprietary code in MatLab r2016b (Mathworks, Natick, Massachusetts, USA) that generated custom image slices. This code used trigonometry based calculations to directly write the slices of the desired object in. PNG file format, rather than starting with a CAD file to slice. The code determined which pixels (micromirrors) would be black (“OFF”), white (“ON”), or grayscale (dithering) based on which pixels fall outside, inside, or on the edge of the desired microneedle part, respectively, in combination with the antialiasing approach used (Additional file 3: Figure S3).

For microneedles without antialiasing, only black (“OFF”) and white (“ON”) pixels were used. When any portion of the desired microneedle array is located on the pixel, the entire pixel would be assigned to completely “ON” (white) in our MatLab program. When no portion of the pixel was covered with the microneedle array, it was assigned to be completely “OFF” (black). No grayscale was utilized in fabricating these needles (Additional file 3: Figure S3).

For microneedles with optimized antialiasing, the grayscale value of the pixel was equivalent to the percentage of that pixel covered by a microneedle. Pixels falling completely inside of a microneedle on the array were white (“ON”), whereas pixels outside of the array were black (“OFF”). Pixels partially covered by the microneedle were grayscale. The light intensity of grayscale pixels was equivalent to the percentage of the micromirror that is covered by the microneedle structure. For example, if a 2 × 2 pixel microneedle were centered on a 3 × 3 pixel array, each side pixel would be projected at 50% of the maximum light intensity and corner pixels would project at 25% of maximum light intensity (Additional file 3: Figure S3).

All microneedles produced using this code had layers measuring 10 μm thick (e.g. a 1000 μm tall microneedle projection would have 100 layers). The system’s default print settings file, which defined exposure time, elevator movement, etc. for 10 μm thick layers and PR48 resin, was combined with all of the slices in a .tar.gz format. The zipped file was loaded onto the Autodesk Ember to fabricate the microneedles.

### Analysis of microneedle scaling

The relationship between the height of the projected microneedle array and the dimensions of the final 3D printed part was also investigated. The height of the projected array was varied from 1000 μm to 1500 μm in height. The MatLab code was used to produce image slices with a 10um layer thickness and optimized antialiasing. Microneedles were then fabricated using Autodesk’s standard clear resin, imaged, and measured as previously described.

### Production of microneedles of varying aspect ratio and spacing

In order to alter the aspect ratio and spacing of the microneedles, the MatLab program was used to directly generate slices of microneedles with various aspect ratios and spacings. To vary aspect ratio, slices of microneedles measuring 450 μm wide and 1450 μm tall, 350 μm wide and 1750 μm tall, and 250 μm wide and 1850 μm tall were generated with optimized antialiasing and 10 μm layer thickness. Edge to edge spacing was equivalent to the width of the microneedle in all cases. To vary spacing, slices of microneedles measuring 1450 μm tall and 450 μm wide were generated with 200 μm, 400 μm and 600 μm spacing (as measured from edge to edge) were generated using optimized antialiasing and 10 μm layer thickness in our custom MatLab code. All microneedles were fabricated using the Autodesk Ember and imaged as previously described.

## Results and discussion

### Fabrication of microneedles using default settings

Square pyramidal microneedle arrays were first 3D printed using the Autodesk’s Print Studio software with varying layer height. The CAD file used to fabricate the array measured 1000 μm in height and 450 μm in width.

**Fig. 3:**
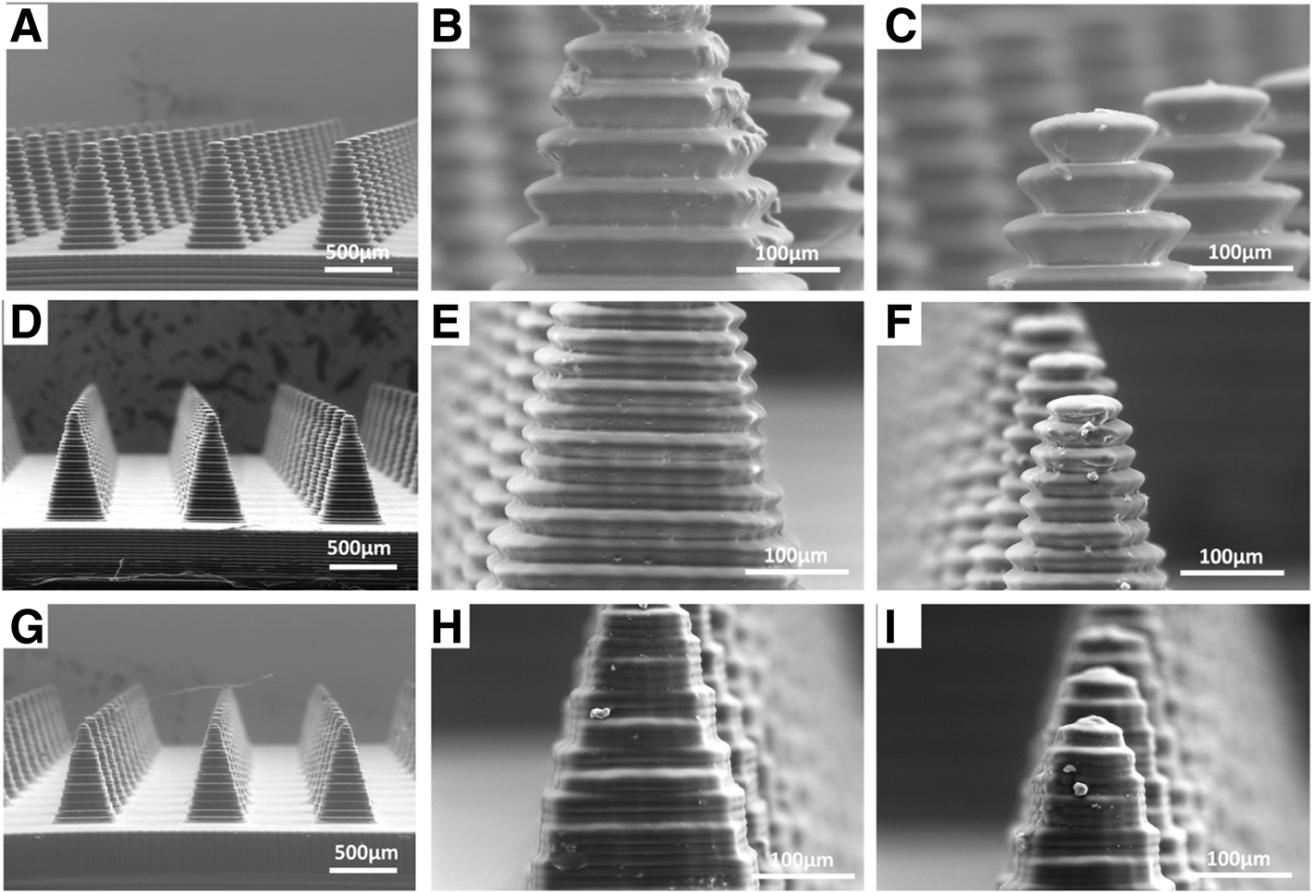
Microneedle masters fabricated using default settings. Microneedle array fabricated using **a-c**) 50 μm layer thickness, **d-f**) 25 μm layer thickness, **g-h**) and 10 μm layer thickness at differing magnifications. Figures **c**), **f**), and **i**) are close-ups of the tips which demonstrate the lack of sharpness which would be required of a needle that could penetrate the skin

Several defects were observed on the 3D printed microneedle array. “Stair-stepping” was visible on all surfaces (Fig. 3a-i), producing a jagged sidewall. This jagged sidewall was aesthetically undesirable and would alter the mechanical properties of the needle. Decreasing layer thickness improved the surface defect. For 50 μm and 25 μm layer heights, “stair-stepping” was present at approximately 50 μm and 25 μm step heights, as expected (Table 1). Interestingly, microneedles sliced into 10 μm layers exhibited both minor stair stepping at 10 μm intervals (as expected) and a larger stepping pattern every 30 μm. Nevertheless, smooth sidewalls were not obtained for any of the available settings.

**Table 1:** Dimensions of microneedles fabricated using default settings. Data are given as mean ± standard deviation (*n* = 6)

Nominal Layer Thickness	Height (μm)	Width (μm)	Tip Radius (μm)	Experimental Layer Thickness (μm)
50 μm	694.9 ± 26.6	450.3 ± 8.6	161.6 ± 14.1	51.1 ± 2.2
25 μm	707.0 ± 4.8	437.2 ± 6.6	40.6 ± 4.6	25.6 ± 1.4
10 μm	711.4 ± 7.3	414.5 ± 17.0	37.9 ± 4.6	10.7 ± 0.8^a^

^a^In addition to this predictable layer thickness, an additional stair-stepping pattern was visualized every three layers

Microneedle sharpness, measured by tip radius of curvature, ranged from ~ 40 μm to 160 μm with thinner layers producing sharper structures. For comparison, microneedle arrays having a tip radius of approximately 20 μm have been shown to effectively penetrate skin [8]. Because the force required to insert a microneedle array into the skin increases with the square of the tip radius, we expect that these Ember microneedles would require very high forces to penetrate skin, if they are able to penetrate at all [21].

Microneedles were shorter than the original CAD file, with a ~ 30% decrease in microneedle height relative to the design, regardless of the slicing and antialiasing settings (Table 1). Microneedle width had close fidelity to the CAD file. Johnson et al. also reported similar findings using the CLIP system [14]. Further, Sun et al. have demonstrated that diffraction and aberration of light introduce predictable defects into parts produced by microstereolithography [19]. The light reflecting off of each micromirror typically spreads into neighboring pixels. Therefore, the amount of light per unit area is greater for large parts (where light from neighboring pixels adds together), as compared to small parts (Additional file 4: Figure S4) [19]. For this reason, small features often fail to cure [19]. We expect that microneedle truncation occured when the structure’s width decreased enough that the light intensity dropped below a minimum threshold for curing. Further experimentation would be required to verify that these previously established phenomena are responsible for the truncation of our microneedle arrays.

### Improvement in microneedle arrays using custom slicing

Next, we investigated the role of aliasing on the defects observed in the default prints. As mentioned previously, aliasing occurs when each slice of a CAD file is converted to an image approximated by a finite number of micromirrors on a DLP chip, which limits the resolution of the image. Anti-aliasing is an approach to artificially improve resolution by introducing grayscale (Fig. 2f) [22].

The slices generated by Print Studio were first visualized to determine whether the default software performs any antialiasing when processing CAD files (Fig. 3c). Inspection of the. PNG image slices showed that some antialiasing is performed, as evidenced by the presence of gray pixels. According to Autodesk, the Autodesk Ember determined grayscale values according to a uniform sampling scheme where each pixel was sampled at 16 different evenly spaced locations, or four locations along each axis (Additional file 3: Figure S3) [23]. The percentage of these sampling locations covered by the desired part determines the grayscale value. For this reason,. PNG image slices generated using PrintStudio had three discrete grayscale values (plus black and white) along each sidewall. Interestingly,. PNG images slices towards the tip of the microneedle always contained nine illuminated pixels, even though the microneedle tip measures less than one pixel wide at the tip. We hypothesize that additional pixels were added by the software to counteract the decrease in light intensity typically associated with small feature sizes (as discussed in the previous section and Additional file 4: Figure S4).

A more optimal approach to antialiasing is to calculate the exact percentage of the pixel area that is covered by the object, rather than sampling at specified locations within the pixel. The light intensity of the pixel in the. PNG image is then equivalent to the calculated percentage. Though this optimized approach is more computationally expensive, it improves quality by incorporating more levels of grayscale.

In order to systematically vary the antialiasing algorithm used, we wrote a program in MatLab to produce custom image files for each layer. Using this program, the light intensity of every pixel in the array was controlled individually. In contrast to the typical workflow where a CAD file is generated, sliced, and converted to an image, our MatLab program directly generated custom slices representing the desired part through mathematical calculations. Three different antialiasing conditions were tested: no antialiasing (Additional file 5: Figure S5), Print Studio’s default antialiasing algorithm (Additional file 6: Figure S6), and an optimized antialiasing algorithm (Additional file 7: Figure S7). See materials and methods for a detailed explanation of each algorithm.

**Fig. 4:**
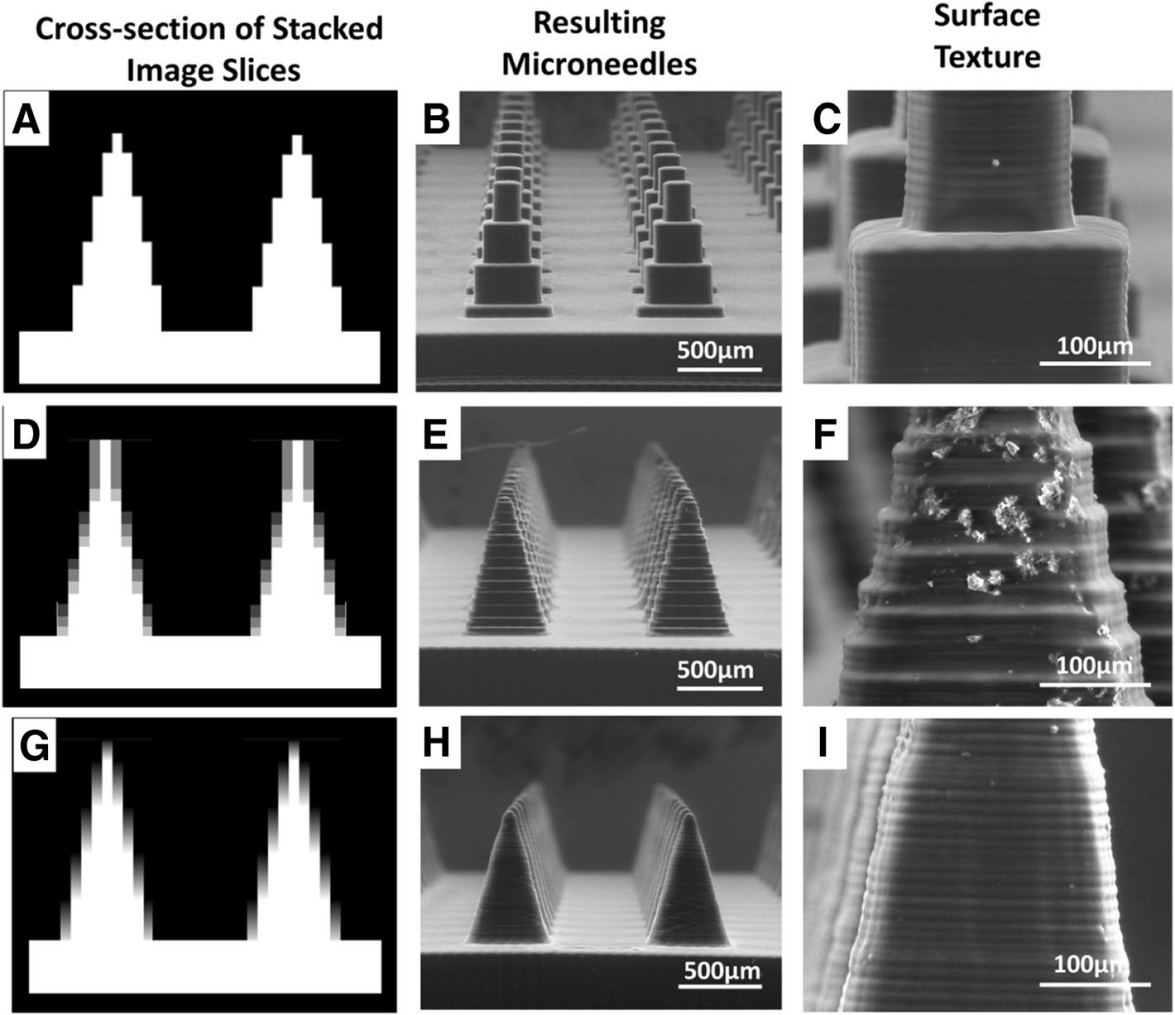
Effect of antialiasing on microneedle geometry **a-c**) Microneedles fabricated without antialiasing **a**) Cross section of a stack of image slices representing two microneedles projected without antialiasing. The cross section is taken at the center of the needle. **b** Low magnification and **c**) high magnification of microneedles fabricated without antialiasing. **d-f** Microneedles fabricated with PrintStudio’s default antialiasing algorithm. **d**) Cross section of a stack of image slices representing two microneedles projected with PrintStudio’s default antialiasing algorithm. The cross section is taken at the center of the needle. **e** Low magnification and **f**) high magnification of microneedles fabricated with PrintStudio’s default antialiasing algorithm. **g-i** Microneedles fabricated with optimized antialiasing **g**) Cross section of a stack of image slices representing two microneedles projected with an optimized antialiasing algorithm. The cross section is taken at the center of the needle. **h** Low magnification and **i**) high magnification of microneedles fabricated with optimized antialiasing

Images of the microneedles fabricated using different antialiasing algorithms demonstrated the importance of antialiasing (Fig. 4). When no antialiasing was used, four discrete steps were visualized. The bottom of the microneedle was 9 × 9 pixels. As the microneedle width narrows moving towards the tip, the object became 7 × 7 pixels, then 5 × 5 pixels, etc. Because every micromirror was either completely “ON” or completely “OFF”, the transition between pixels was abrupt; the height of each layer corresponded to the transition between pixels. Print Studio’s default antialiasing algorithm improved the structure (Fig. 4e) as compared to the structure without antialiasing (Fig. 4b). Using Print Studio’s algorithm, distinct layers were visible both every 10 μm, due to the layer height and movement of the build elevator, and at every 30 μm due to the transition between different levels of gray scale (which corresponded to transitions between different projected light intensities). The introduction of an optimized antialiasing algorithm though our custom MatLab program further improved the surface roughness of the microneedle sidewall (Fig. 4h-i). Here, only the 10 μm layers were present. Surface roughness was barely perceptible by electron microscopy.

### Analysis and correction of microneedle dimensions

**Fig. 5:**
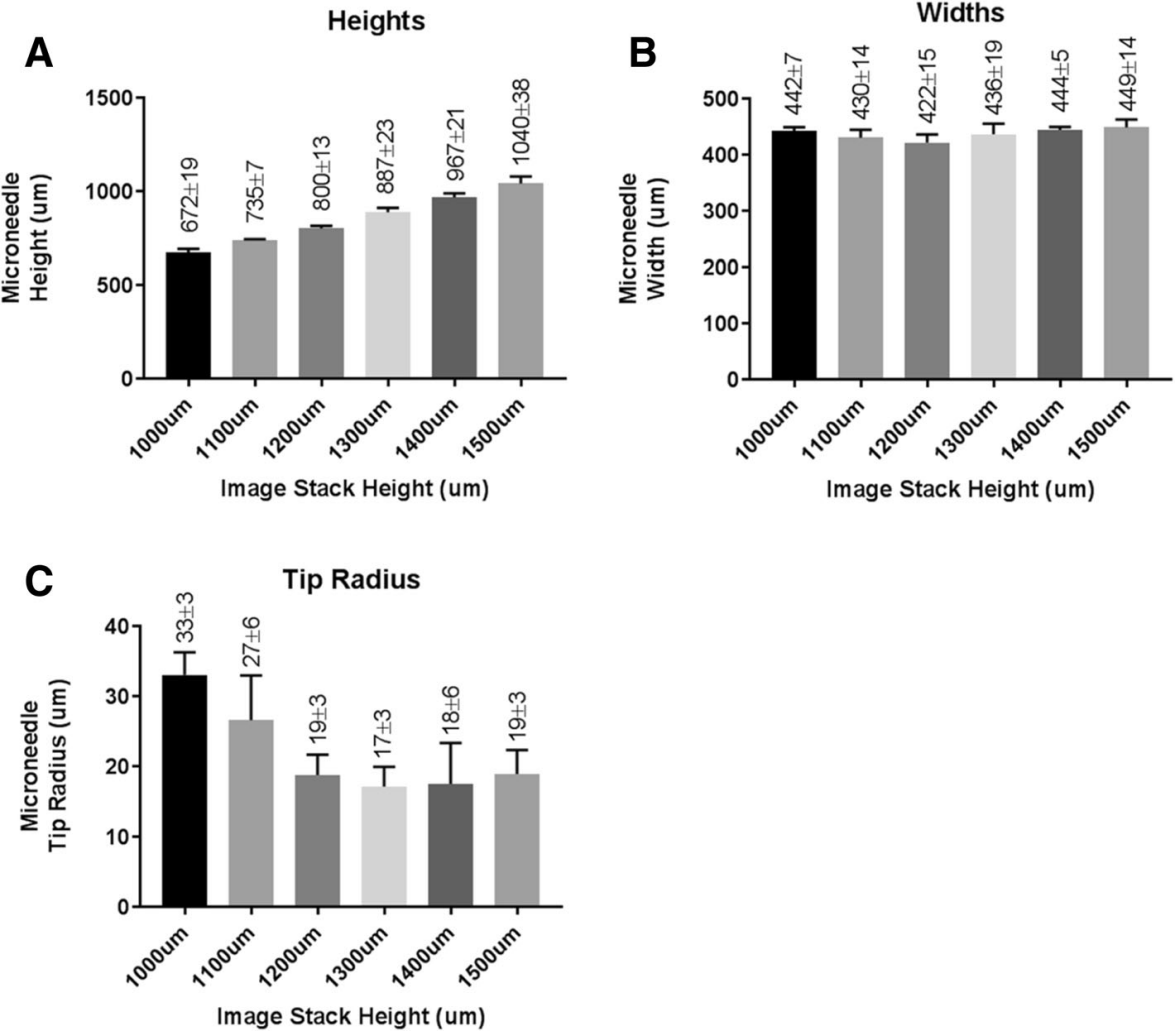
Effect of image stack height on microneedle dimensions. Impact of the image stack height on microneedle **a**) height, **b**) width and **c**) tip radius

Fundamental properties of light such as diffraction are known to alter the dimensions of small 3D printed parts [15, 20]. Therefore, the dimensions of the 3D printed microneedle arrays were analyzed (Fig. 5). As previously mentioned, microneedles were found to be approximately 30% shorter than the intended height of 1000 μm, but similar to the intended width of 450 μm.

The height of the image stack was then increased (to project a microneedle measuring between 1100 and 1500 μm in height) to counteract truncation. As expected, increasing the height of the image stack increased the height of the resulting microneedle. When an appropriate image stack height was selected (between 1400 and 1500 μm), the intended microneedle height of approximately 1000 μm (within 5% error) was achieved. Again, no changes in microneedle width were observed with changes in the image stack height, as expected.

Interestingly, microneedle sharpness (as measured by tip radius of curvature) did change with image stack height. As the aspect ratio (the ratio of the height to the width of the needle) increased, the tip radius decreased, producing sharper microneedles. We hypothesize that increased slope of the sidewall allows the system to more slowly approach the minimum curable feature width, producing a sharper tip. The maximum sharpness achieved was a tip radius of less than 20 μm, which is consistent with other microneedles that successfully penetrate skin. [8]

### Ability to adjust microneedle aspect ratio and spacing

**Fig. 6:**
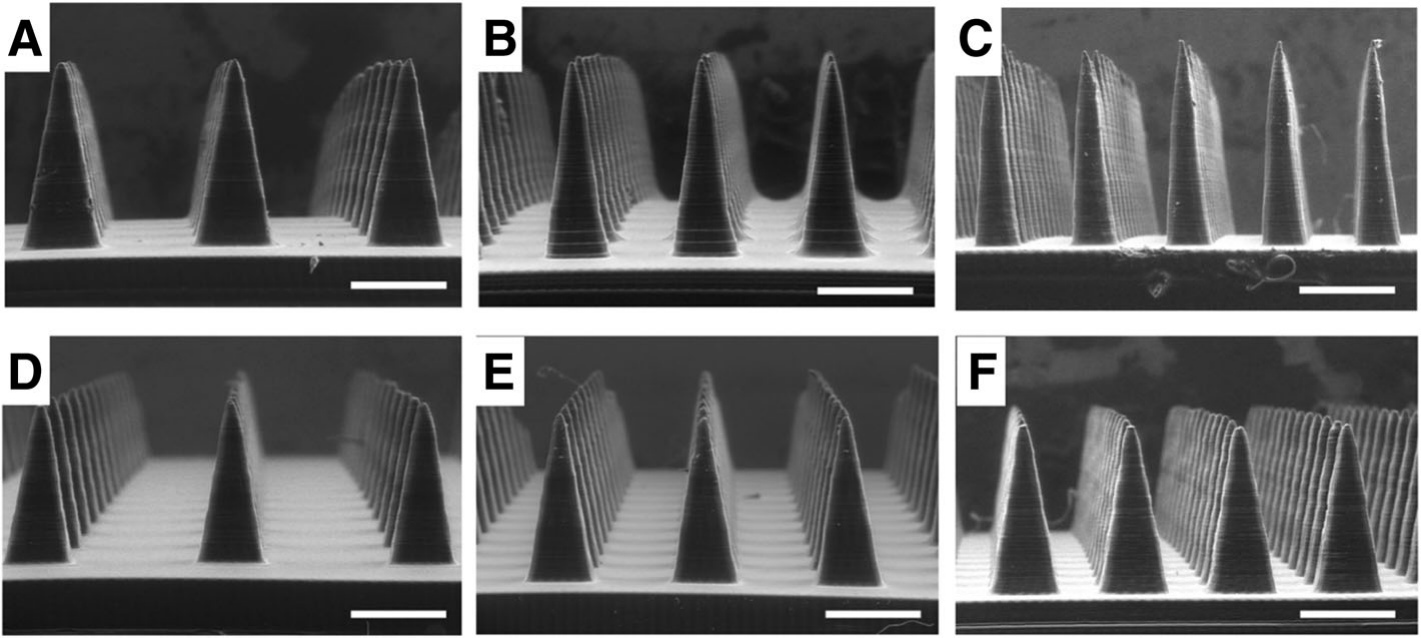
Microneedles of varying aspect ratios and spacings. **a-c** Microneedles measuring approximatley 1000 μm in height with widths of approximately **a**) 450 μm, **b**) 350 μm and **c**) 250 μm. **d-f** Microneedles measuring approximately 1000 μm in height and 450 μm in width with interneedle spacing of **d**) 600 μm, **e**) 400 μm and **f**)200 μm, respectively, as measured from edge to edge

An important advantage of 3D printing microneedle masters is the ability to rapidly customize microneedle design. Microneedle aspect ratio and spacing are important design parameters that affect microneedle strength and total possible drug loading. To demonstrate the ability to easily adjust microneedle shape though 3D printing, we used our MatLab program and the Autodesk Ember to produce microneedles having widths that vary between 250 μm and 450 μm (Fig. 6a-c, Table 2) with a constant height of approximately 1000 μm. The actual dimensions of the microneedles were found to be within ±10% of the intended dimensions in all cases. Further improvements in the fidelity of actual dimensions to the intended dimensions could be achieved by optimizing scaling, as demonstrated in the previous section. Equivalent methods were used to fabricate microneedles with interneedle spacing varying between 200 μm and 600 μm, as shown in Fig. 6d-f. These microneedles measure approximately 1000 μm in height and 450 μm in width. Therefore, the Autodesk Ember enables microneedles of varying geometries to be readily fabricated.

**Table 2:** Dimensions of microneedles of different aspect ratios. Data are given as mean ± standard deviation (*n* = 6)

Figure	Height (μm)	Width (μm)	Tip Radius (μm)
6A	937.7 ± 43.8	433.0 ± 13.2	17.1 ± 6.7
6B	1096.5 ± 33.7	355.4 ± 3.6	12.0 ± 2.3
6C	1046.0 ± 39.5	253.7 ± 6.2	9.5 ± 1.2

## Conclusions

In summary, we demonstrate a simple and low cost method for fabricating microneedle masters using a desktop 3D printer. The printer’s default settings introduce defects into the fabricated microneedles, but proper optimization using a combination of reducing layer height, employing a high quality antialiasing algorithm, and rescaling the input images enables high quality microneedles to be produced. The microneedles demonstrate sharp tip radii with fabrication times less than one hour. Further, we demonstrate that the height, width and spacing of these microneedle masters can be easily adjusted to optimize microneedle design. Though only one photopolymerizable resin was used in this work, we anticipate that the approaches outlined in this article would be generalizable to other 3D printing resins [14]. Microneedle masters produced using this technique could also be combined with standard silicone micromolding approaches to fabricate microneedles from desired non-photopolymerizable materials, such as water soluble or biodegradable polymers mixed with therapeutic agents. We anticipate that this approach will lower the barrier to entry into the microneedle field for researchers with little existing equipment or a modest background in microfabrication and provide an easy way to adjust key microneedle parameters, such as size, aspect ratio, and spacing.

## Additional files

Additional file 1: **Figure S1**. Image of microneedle CAD file created in Solidworks® 2016. (DOCX 478 kb)

Additional file 2: **Figure S2**. Autodesk Ember PR48 resin formulation. Figure reproduced with permission from Autodesk. (DOCX 192 kb)

Additional file 3: **Figure S3**. Explanation of antialiasing algorithms. A) A slice of a single microneedle on an array of pixels. The red dotted line is a microneedle and each white square is a pixel. B) When optimized antialiasing is used, the light intensity of each pixel is equivalent to the percent of the pixel area that is covered by the microneedle C) When PrintStudio’s default antialiasing algorithm is used, each pixel is sampled at sixteen locations. The light intenisty of the pixel is equivalent to the percentage of those sampling locations that are covered by the microneedle. D) When no antialiasing is used, the pixel is ON if any portion of the microneedle falls on the pixel. (DOCX 487 kb)

Additional file 4: **Figure S4**. Light effects at small feature sizes. A) When a single 50 μm pixel is projected onto the build area, diffraction and aberration of the light cause the light to be wider than 50 μm at the build surface. B) Therefore, light projections from neighboring pixels overlap. The additive effect of overlapping light from neighboring pixels cause C) the maximum light intensity projected off of a single pixel to be less than D) the maximul light intenisty resulting from two neighboring pixels. E) Therefore, the projected light intensity increases as a function of feature width. (DOCX 132 kb)

Additional file 5: **Figure S5**. Image slices output from Matlab code without antialiasing. Image slices for a single microneedle on layers 1 through 15 and layers 91 through 100 when no antialiasing algorithm is used. Note that slices 16–90 are omitted due to space constraints and that slice numbers begin at the first slice of the microneedle, not the first slice of the base of the array. (DOCX 199 kb)

Additional file 6: **Figure S6**. PNG Image slices from Printstudio’s default antialiasing. Image slices for a single microneedle on layers 1 through 15 and layers 91 through 100 when PrintStudio’s default antialiasing algorithm is used. Note that slices 16–90 are omitted due to space constraints and that slice numbers begin at the first slice of the microneedle, not the first slice of the base of the array. (DOCX 224 kb)

Additional file 7: **Figure S7**. Image slices output from Matlab code with optimized antialiasing. Image slices for a single microneedle on layers 1 through 10 and layers 91 through 100 when the optimized antialiasing algorithm is used. Note that slices 11–90 are omitted due to space constraints and that slice numbers begin at the first slice of the microneedle, not the first slice of the base of the array. (DOCX 216 kb)

## Abbreviations

3D: Three dimensional
CAD: Computer aided design
CLIP: Continuous Liquid Interface Production
DLP: Digital light projection
kV: Kilovolts
PNG: Portable network graphic
STL: Standard triangle language
USA: United States of America
USD: United States dollars
μm: Micrometer
