# Supplementary figures and images for: Low cost additive manufacturing of microneedle masters
Source: 3D Print Med. 2019 Feb 4;5:2. doi: 10.1186/s41205-019-0039-x (PMC6676342; doi:10.1186/s41205-019-0039-x)

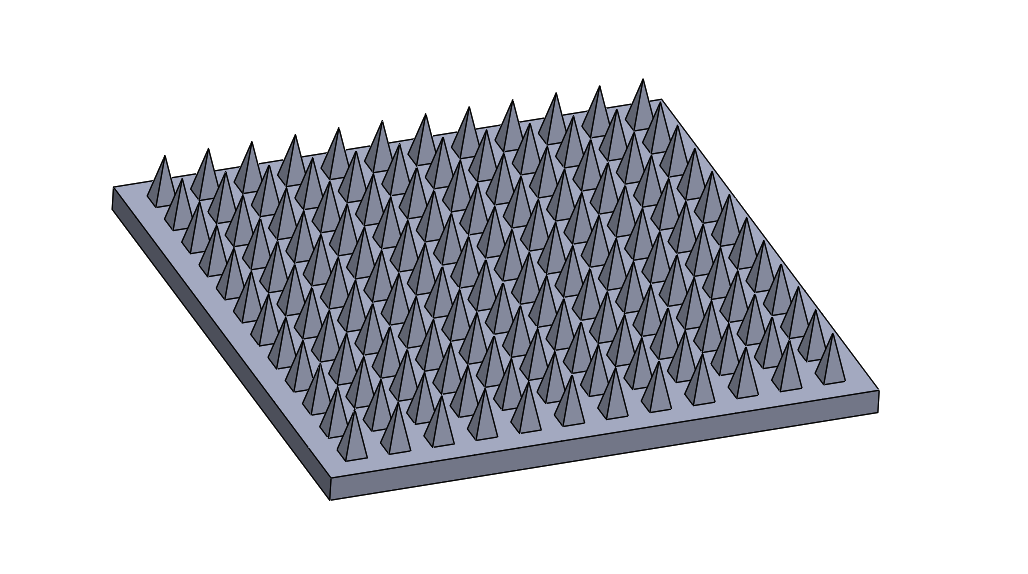


**Additional File 1. Image of microneedle CAD file created in Solidworks® 2016.**

Supplement: Supplementary file 1 — Figure S1. Image of microneedle CAD file created in Solidworks® 2016. (DOCX 478 kb) [file 41205_2019_39_MOESM1_ESM.docx]
