# Supplementary material for: Low cost additive manufacturing of microneedle masters
Source: 3D Print Med. 2019 Feb 4;5:2. doi: 10.1186/s41205-019-0039-x (PMC6676342; doi:10.1186/s41205-019-0039-x)
Supplement: Supplementary file 2 — Figure S2. Autodesk Ember PR48 resin formulation. Figure reproduced with permission from Autodesk. (DOCX 192 kb) [file 41205_2019_39_MOESM2_ESM.docx]

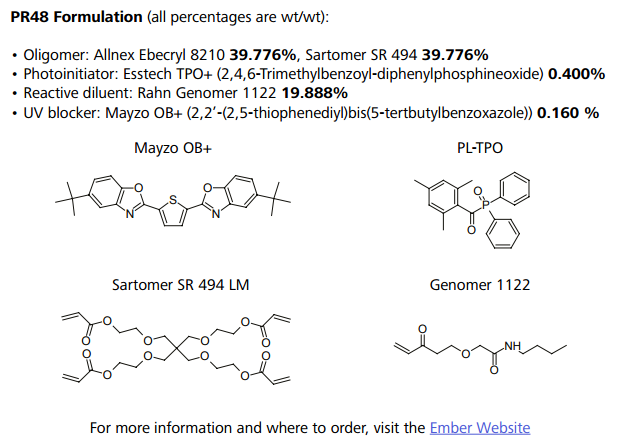


**Additional File 2. Autodesk Ember PR48 resin formulation.** Figure reproduced with permission from Autodesk.
