# Supplementary material for: Low cost additive manufacturing of microneedle masters
Source: 3D Print Med. 2019 Feb 4;5:2. doi: 10.1186/s41205-019-0039-x (PMC6676342; doi:10.1186/s41205-019-0039-x)
Supplement: Supplementary file 3 — Figure S3. Explanation of antialiasing algorithms. A) A slice of a single microneedle on an array of pixels. The red dotted line is a microneedle and each white square is a pixel. B) When optimized antialiasing is used, the light intensity of each pixel is equivalent to the percent of the pixel area that is covered by the microneedle C) When PrintStudio’s default antialiasing algorithm is used, each pixel is sampled at sixteen locations. The light intenisty of the pixel is equivalent to the percentage of those sampling locations that are covered by the microneedle. D) When no antialiasing is used, the pixel is ON if any portion of the microneedle falls on the pixel. (DOCX 487 kb) [file 41205_2019_39_MOESM3_ESM.docx]

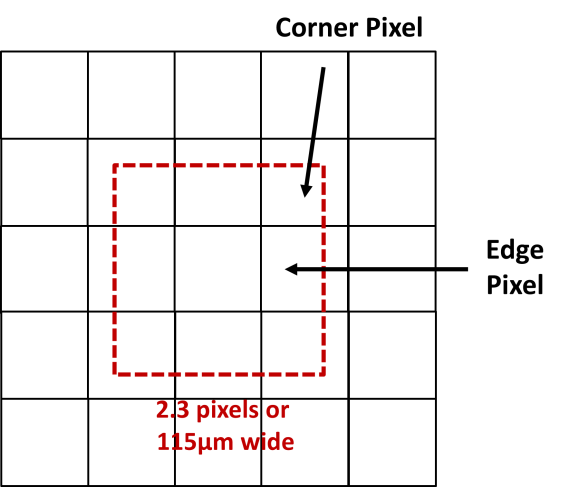

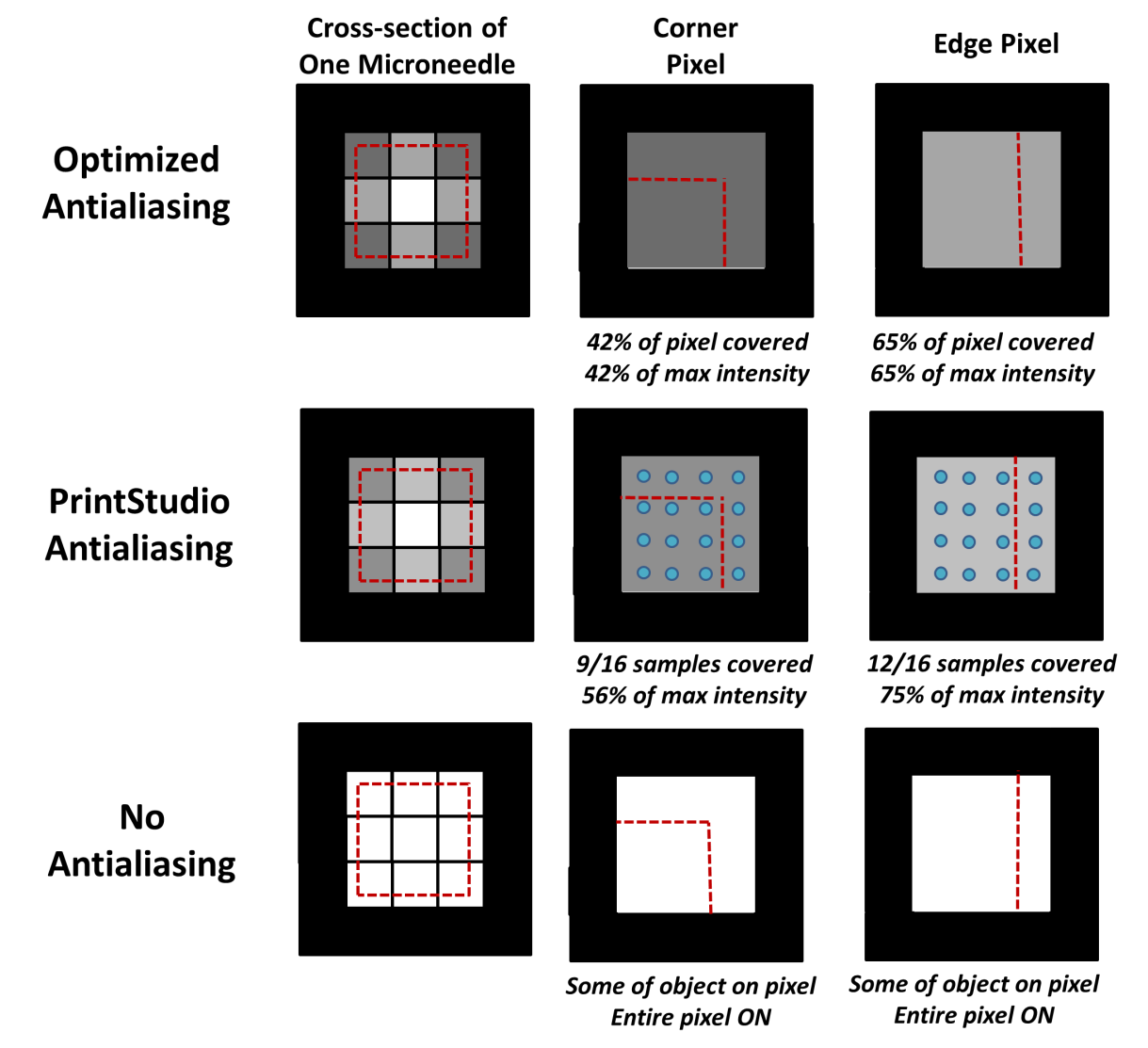


**A**

**B**

**C**

**D**

**Additional File 3. Explanation of antialiasing algorithms.** A) A slice of a single microneedle on an array of pixels. The red dotted line is a microneedle and each white square is a pixel. B) When optimized antialiasing is used, the light intensity of each pixel is equivalent to the percent of the pixel area that is covered by the microneedle C) When PrintStudio’s default antialiasing algorithm is used, each pixel is sampled at sixteen locations. The light intensity of the pixel is equivalent to the percentage of those sampling locations that are covered by the microneedle. D) When no antialiasing is used, the pixel is ON if any portion of the microneedle falls on the pixel.
