# Supplementary material for: Low cost additive manufacturing of microneedle masters
Source: 3D Print Med. 2019 Feb 4;5:2. doi: 10.1186/s41205-019-0039-x (PMC6676342; doi:10.1186/s41205-019-0039-x)
Supplement: Supplementary file 4 — Figure S4. Light effects at small feature sizes. A) When a single 50 μm pixel is projected onto the build area, diffraction and aberration of the light cause the light to be wider than 50 μm at the build surface. B) Therefore, light projections from neighboring pixels overlap. The additive effect of overlapping light from neighboring pixels cause C) the maximum light intensity projected off of a single pixel to be less than D) the maximul light intenisty resulting from two neighboring pixels. E) Therefore, the projected light intensity increases as a function of feature width. (DOCX 132 kb) [file 41205_2019_39_MOESM4_ESM.docx]

**
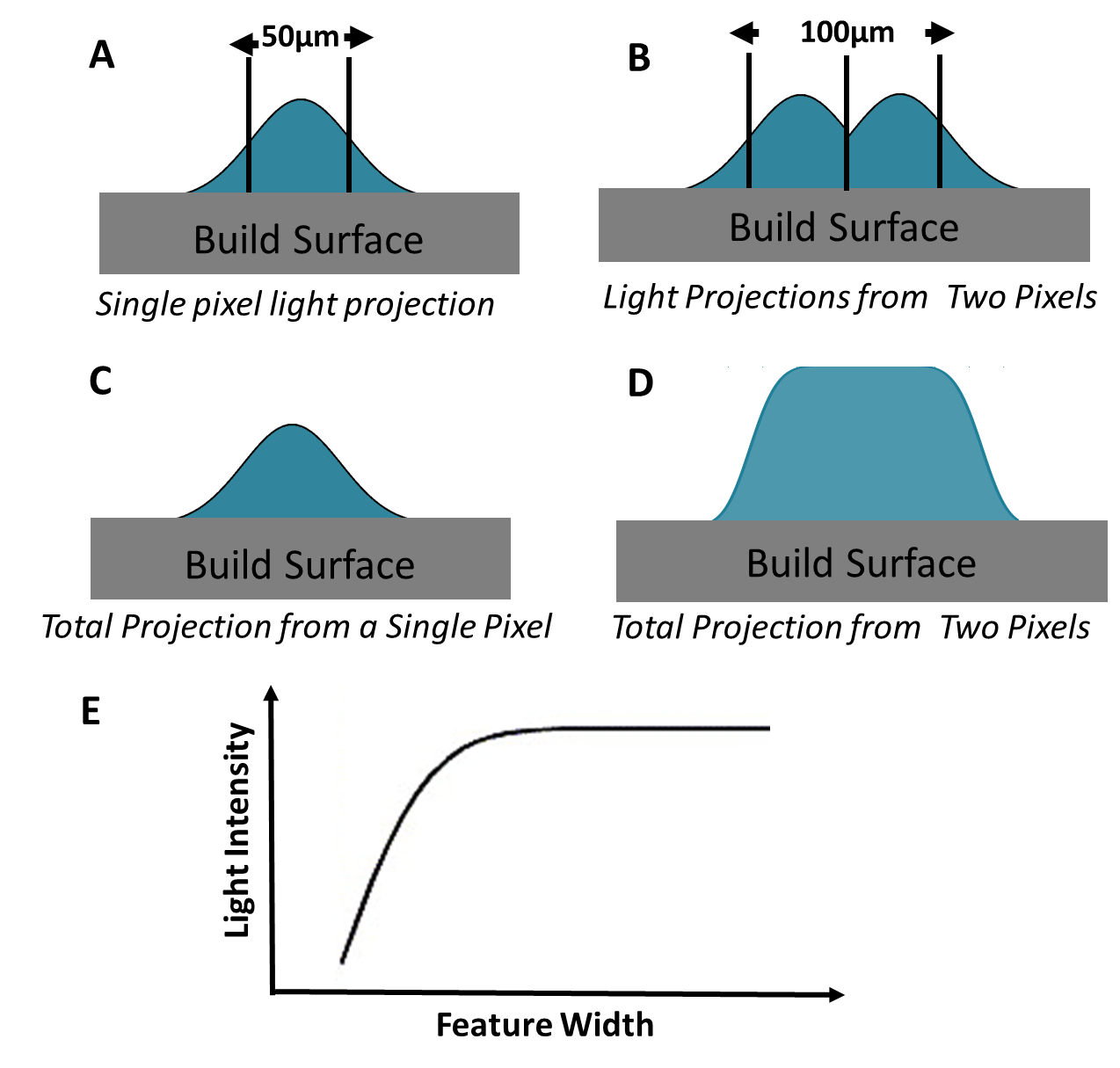
**

**Additional File 4. Light effects at small feature sizes.** A) When a single 50µm pixel is projected onto the build area, diffraction and aberration of the light cause the light to be wider than 50µm at the build surface. B) Therefore, light projections from neighboring pixels overlap. The additive effect of overlapping light from neighboring pixels cause C) the maximum light intensity projected off of a single pixel to be less than D) the maximum light intensity resulting from two neighboring pixels. E) Therefore, the projected light intensity increases as a function of feature width.
