# Supplementary material for: Low cost additive manufacturing of microneedle masters
Source: 3D Print Med. 2019 Feb 4;5:2. doi: 10.1186/s41205-019-0039-x (PMC6676342; doi:10.1186/s41205-019-0039-x)
Supplement: Supplementary file 5 — Figure S5. Image slices output from Matlab code without antialiasing. Image slices for a single microneedle on layers 1 through 15 and layers 91 through 100 when no antialiasing algorithm is used. Note that slices 16–90 are omitted due to space constraints and that slice numbers begin at the first slice of the microneedle, not the first slice of the base of the array. (DOCX 199 kb) [file 41205_2019_39_MOESM5_ESM.docx]

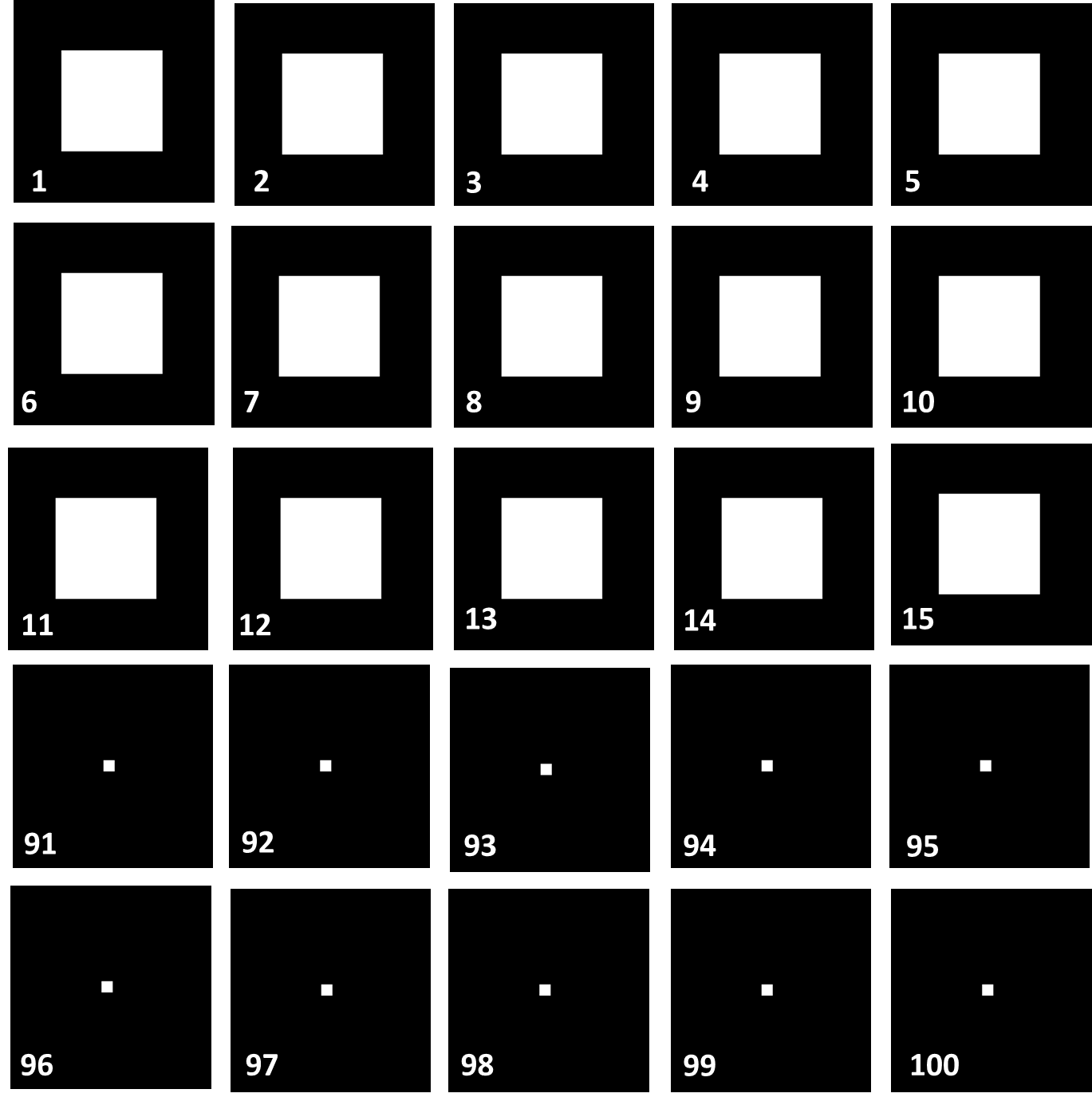


Additional File 5. Image slices output from Matlab code without antialiasing. Image slices for a single microneedle on layers 1 through 15 and layers 91 through 100 when no antialiasing algorithm is used. Note that slices 16-90 are omitted due to space constraints and that slice numbers begin at the first slice of the microneedle, not the first slice of the base of the array.
