# Supplementary material for: Low cost additive manufacturing of microneedle masters
Source: 3D Print Med. 2019 Feb 4;5:2. doi: 10.1186/s41205-019-0039-x (PMC6676342; doi:10.1186/s41205-019-0039-x)
Supplement: Supplementary file 6 — Figure S6. PNG Image slices from Printstudio’s default antialiasing. Image slices for a single microneedle on layers 1 through 15 and layers 91 through 100 when PrintStudio’s default antialiasing algorithm is used. Note that slices 16–90 are omitted due to space constraints and that slice numbers begin at the first slice of the microneedle, not the first slice of the base of the array. (DOCX 224 kb) [file 41205_2019_39_MOESM6_ESM.docx]

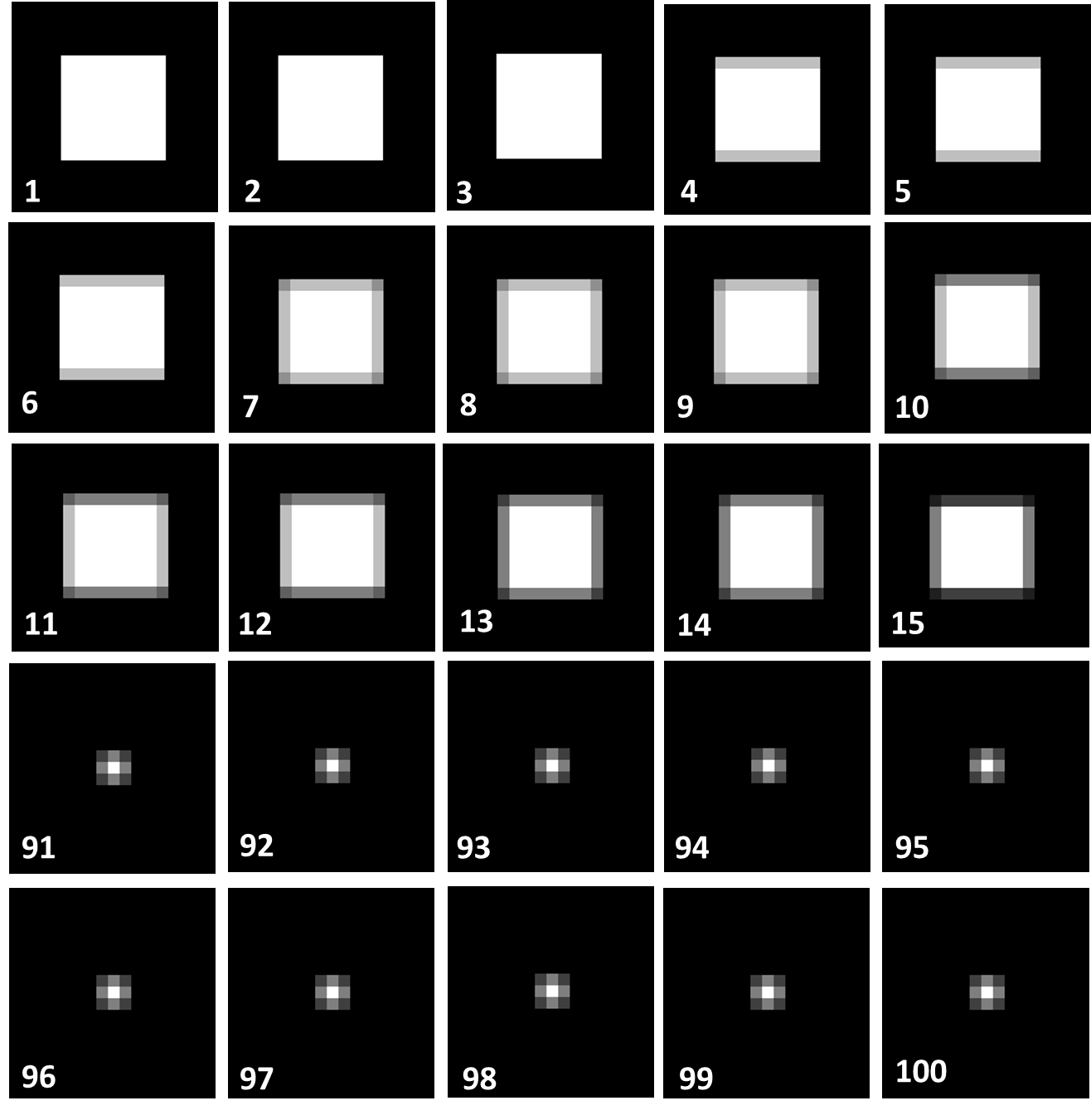


Additional File 6. PNG Image slices from Printstudio’s default antialiasing. Image slices for a single microneedle on layers 1 through 15 and layers 91 through 100 when PrintStudio’s default antialiasing algorithm is used. Note that slices 16-90 are omitted due to space constraints and that slice numbers begin at the first slice of the microneedle, not the first slice of the base of the array.
