# Supplementary material for: Low cost additive manufacturing of microneedle masters
Source: 3D Print Med. 2019 Feb 4;5:2. doi: 10.1186/s41205-019-0039-x (PMC6676342; doi:10.1186/s41205-019-0039-x)
Supplement: Supplementary file 7 — Figure S7. Image slices output from Matlab code with optimized antialiasing. Image slices for a single microneedle on layers 1 through 10 and layers 91 through 100 when the optimized antialiasing algorithm is used. Note that slices 11–90 are omitted due to space constraints and that slice numbers begin at the first slice of the microneedle, not the first slice of the base of the array. (DOCX 216 kb) [file 41205_2019_39_MOESM7_ESM.docx]

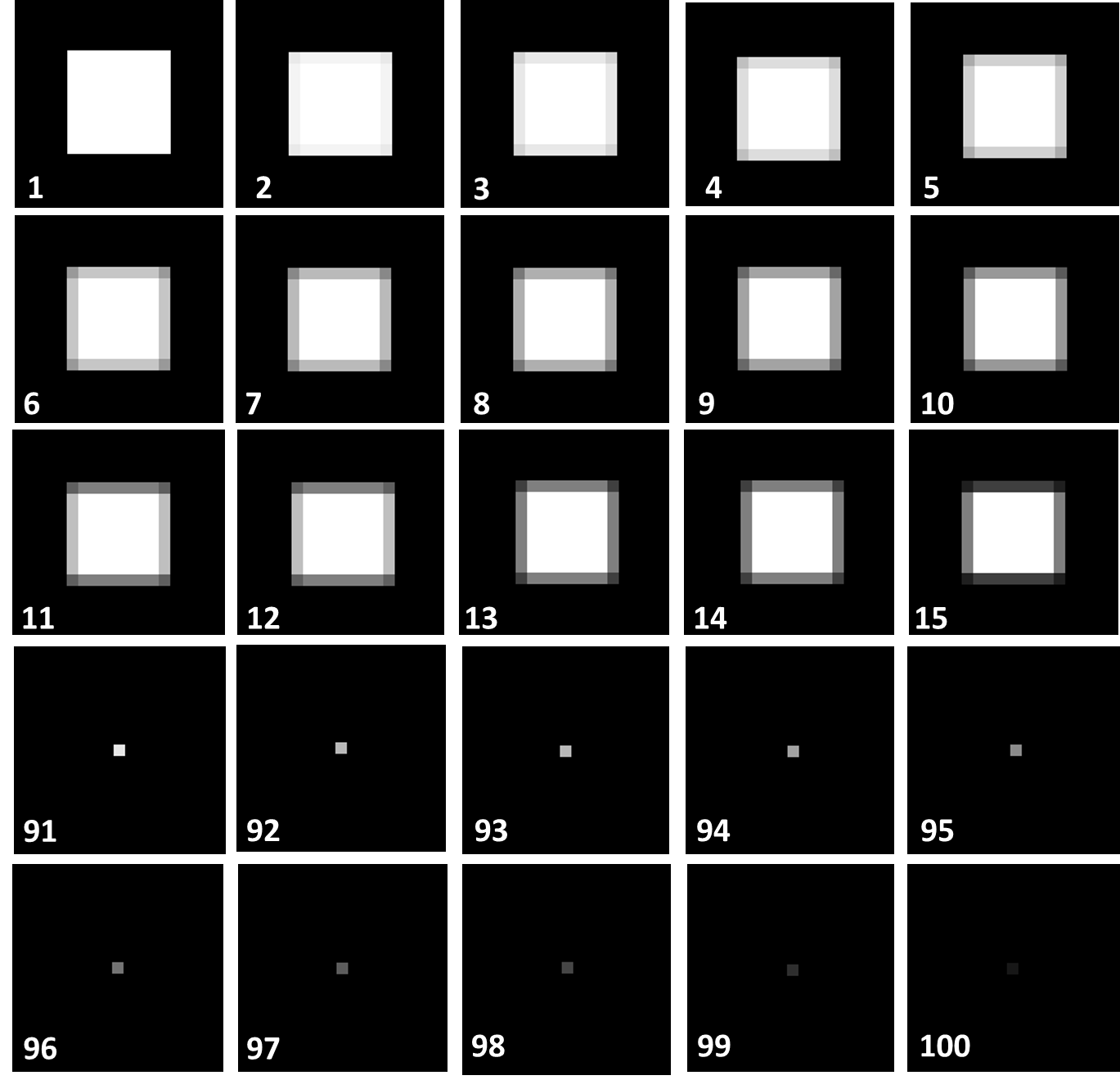


Additional File 7. Image slices output from Matlab code with optimized antialiasing. Image slices for a single microneedle on layers 1 through 10 and layers 91 through 100 when the optimized antialiasing algorithm is used. Note that slices 11-90 are omitted due to space constraints and that slice numbers begin at the first slice of the microneedle, not the first slice of the base of the array.
